# Response to comment on: Gleicher N et al., 2016. Reprod biol endocrinol Sep 5;14(1):54

**DOI:** 10.1186/s12958-017-0241-x

**Published:** 2017-04-05

**Authors:** Norbert Gleicher, Andrea Vidali, Jeffrey Braverman, Vitaly A. Kushnir, David H. Barad, Cynthia Hudson, Yang-Guan Wu, Qi Wang, Lin Zhang

**Affiliations:** 1 The Center for Reproduction, 21 E 69th St, New York, NY 10021 USA; 2 The Foundation for Reproductive Medicine, New York, USA; 3 The Brivanlou Laboratory for Stem Cell Biology and Molecular Embryology, Rockefeller University, New York, NY USA; 4 Medical University of Vienna, Vienna, Austria; 5 Fertility Specialist in New York, New York, NY USA; 6 Braverman IVF and Reproductive Immunology, New York, NY USA; 7 Wake Forest University, Winston Salem, NC USA

**Keywords:** Preimplantation genetic screening (PGS), In vitro fertilization (IVF), Embryos, Embryo mosaicism, Trophectoderm biopsy, Blastocyst

We appreciate the interest of Tiegs et al. in our manuscript [1] but find the criticism surprising. When we wrote our paper, their abstract had been published in 2015 in *Fertility&Sterility* [2]. It, therefore, was appropriately referenced, while their full length manuscript, in contrast, had not yet been published and, therefore, was at the time unknown to us. The manuscript appeared only at the end of July [3]. Had we known of the manuscript we, of course, would have quoted the full length manuscript, rather than the abstract, though both in the end offered the same data set, out of a total of 525 embryo transfers, reporting on only 5 alleged clinical misdiagnoses that were reanalyzed.

It is important to note that, therefore, only these 5 cases (and not 525 cases) underwent a second preimplantation genetic screening (PGS) testing round, thereby offering the opportunity to reevaluate only these cases for the possible presence of mosaicism. It is also noteworthy that this repeat testing was done with the same testing platform as before, array genomic hybridization (aCGH), which since has been declared “inadequate” for PGS testing by the PGD International Society (PGDIS) since aCGH cannot reliably detect mosaicism [4]. We will further discuss this fact below in more detail. Based on the repeat testing round, Tiegs et al. concluded that 2/5 embryos (40%) were mosaic because repeat testing gave different results.

The remaining 520 embryos were, however, not reexamined. To, therefore, suggest that the mosaicism rate was 2/525 rather than 2/5 is incorrect. The authors, of course, have no way of knowing to what degree results of a second testing round of these 520 embryos would have differed from the first round.

As the results of our study demonstrated, even within the same PGS laboratory multiple embryo biopsies from same embryos diverged in approximately 50 percent of embryos [1]. Others reported similar outcomes from multiple biopsies [5], and even the PGDIS has acknowledged this fact [4].

We, of course, do acknowledge that five embryos represent a small sample size but, until publication of our study [1], the literature really offered almost no data on embryos undergoing repeat and/or multiple biopsies. Even our study only reported on 11 embryos that underwent repeat biopsies. Tiegs et al., however, factually misrepresented our manuscript in their letter to the editors when claiming that we failed to point out that their study included only five cases. Very much to the contrary, this is exactly what we did when writing: “*The small number of evaluated embryos* (referring to our study) *does not allow ultimate conclusions about what likely mosaicism rates in human embryos may be. Here presented data do, however, suggest that they are significantly higher than the 4.8% rate recently detected by Greco* et al. [reference] *and more in line with the 2/5 (40%) recently reported by Tiegs* et al. [Reference].” We, thus, very clearly pointed out the small number of cases in their report.

Because the basic concept of PGS is being questioned on theoretical, experimental and clinical grounds, our specialty is currently in the midst of an at times unfortunately vitriolic dispute about the clinical value of PGS. The work of our, an Italian [6] and an Israeli group [5] basically forced the PGDIS in July of this year to radically redefine how PGS results should be reported out [4]. In revised guidelines the PGDIS fully acknowledges that mosaicism at blastocyst stage is apparently much more frequent than has previously been acknowledged. The society, however, also points out that the only platform that should be used in PGS is NGS (Next Generation Sequencing) since, it alone, can detect mosaicism above a 20% threshold in a single trophectoderm biopsy. In other words, aCGH (used in the study by Tiegs et al.), has been disavowed by the society because it underestimates mosaicism rates.

Moreover, the society now defines up to 80% mosaicism in a single trophectoderm biopsy as “normal mosaic,” establishing such embryos, therefore, as potentially transferrable. This alone demonstrates that the claim by Tiegs et al. of a 0.7% mosaicism rate in trophectoderm biopsies is unsustainable. That they make this claim based on newborn testing also demonstrates that they have not taken into consideration recent data that demonstrate the incredible ability of (at least rodent) embryos to self-correct downstream from blastocyst stage [7]. Moreover they, likely also did not consider that the trophectoderm, from which biopsies are taken for PGS, becomes the placenta, which for decades has been known to be riddled with aneuploid clonal cell island and, therefore, very obviously is mosaic, even if the fetus, arising from the inner cell mass, is not.

All of these data suggest that Tiegs et al. really have to reconsider conclusions of their abstract [2] and manuscript [3].
